# The interplay between neoantigens and immune cells in sarcomas treated with checkpoint inhibition

**DOI:** 10.3389/fimmu.2023.1226445

**Published:** 2023-09-20

**Authors:** Irantzu Anzar, Brandon Malone, Pubudu Samarakoon, Ioannis Vardaxis, Boris Simovski, Hugues Fontenelle, Leonardo A. Meza-Zepeda, Richard Stratford, Emily Z. Keung, Melissa Burgess, Hussein A. Tawbi, Ola Myklebost, Trevor Clancy

**Affiliations:** ^1^ Oslo Cancer Cluster, NEC OncoImmunity AS, Oslo, Norway; ^2^ Institute of Clinical Medicine, University of Oslo, Oslo, Norway; ^3^ Institute for Cancer Research, Oslo University Hospital, Oslo, Norway; ^4^ Genomics Core Facility, Department of Core Facilities, Oslo University Hospital, Oslo, Norway; ^5^ Department of Surgical Oncology, The University of Texas MD Anderson Cancer Center, Houston, TX, United States; ^6^ Department of Medical Oncology, University of Pittsburgh Medical Center, Pittsburgh, PA, United States; ^7^ Department of Melanoma Medical Oncology, The University of Texas MD Anderson Cancer Center, Houston, TX, United States; ^8^ Department of Clinical Science, University of Bergen, Bergen, Norway

**Keywords:** neoantigens, checkpoint inhibition therapy, next generation sequencing, biomarker discovery, machine learning, immune escape

## Abstract

**Introduction:**

Sarcomas are comprised of diverse bone and connective tissue tumors with few effective therapeutic options for locally advanced unresectable and/or metastatic disease. Recent advances in immunotherapy, in particular immune checkpoint inhibition (ICI), have shown promising outcomes in several cancer indications. Unfortunately, ICI therapy has provided only modest clinical responses and seems moderately effective in a subset of the diverse subtypes.

**Methods:**

To explore the immune parameters governing ICI therapy resistance or immune escape, we performed whole exome sequencing (WES) on tumors and their matched normal blood, in addition to RNA-seq from tumors of 31 sarcoma patients treated with pembrolizumab. We used advanced computational methods to investigate key immune properties, such as neoantigens and immune cell composition in the tumor microenvironment (TME).

**Results:**

A multifactorial analysis suggested that expression of high quality neoantigens in the context of specific immune cells in the TME are key prognostic markers of progression-free survival (PFS). The presence of several types of immune cells, including T cells, B cells and macrophages, in the TME were associated with improved PFS. Importantly, we also found the presence of both CD8+ T cells and neoantigens together was associated with improved survival compared to the presence of CD8+ T cells or neoantigens alone. Interestingly, this trend was not identified with the combined presence of CD8+ T cells and TMB; suggesting that a combined CD8+ T cell and neoantigen effect on PFS was important.

**Discussion:**

The outcome of this study may inform future trials that may lead to improved outcomes for sarcoma patients treated with ICI.

## Introduction

Sarcomas are a rare and heterogenous group of tumors that account for 1% of all cancers and 10-15% of solid tumors in children and young adults (1, 2). They are generally divided into soft tissue sarcomas (STS) and bone sarcomas (BS); however, sarcomas are effectively comprised of more than 150 distinct subtypes (1, 3). The majority of sarcomas are STS, while BS accounts for 15% of cases (2). In addition to the rarity of sarcomas in adults, each distinct subtype is associated with diverse genetic, molecular, anatomical, clinical and/or age related factors; making their study, diagnosis or treatment enormously challenging (4). Sarcomas are broadly considered to be “cold” tumors, with low immune cell infiltration, making them potentially challenging targets of ICI therapy (5).

Although sarcomas have had poor performance in ICI therapy clinical trials (5), the tremendous success of ICI in other cancer indications offers prospects that a path toward curative immunotherapy success may be possible for sarcoma patients. Several clinical trials evaluating ICI in sarcomas (including the SARC028/NCT02301039 trial pertaining to this study) have reported at best only modest responses, with an overall response rate (ORR) of approximately 15% (6–10). However, a small number of patients in these clinical studies had a notable positive clinical outcome. This observation, coupled with the complete remissions reported in individual case reports, warrants further research to decipher the precise mechanisms of the positive ICI therapy responses in sarcomas (11, 12), and potentially to develop predictive biomarkers for ICI and other cancer immunotherapies (6, 9, 13, 14). The tumor mutational burden (TMB), conventionally used as a predictive biomarker to ICI therapy in several cancer types (15), is an obvious immunogenomic property to investigate and potentially help elucidate the mechanisms associated to a positive clinical response. However, as we will report here and as reported in previously published studies, TMB remains inconclusive as a biomarker for ICI therapy response in sarcomas (11, 16). Therefore, in this study we were motivated to study the interplay between multiple immunogenomic properties in addition to TMB, such as neoantigen load and infiltration of several immune-cell types into the TME (11, 12).

To achieve this, we investigated 31 available samples from ICI treated sarcoma patients in the SARC028 clinical trial (6). We performed whole-exome sequencing (WES) of tumor and matched normal blood from patients and RNA-sequencing (RNA-seq) of their tumors and identified neoantigens corresponding to multiple sources of genomic variants including single nucleotide variants (SNVs), small insertion and deletions, and gene fusion events. The RNA-seq data from their tumors was used to characterize immune cell infiltration into the TME and the expression patterns of immune-related genes to improve our understanding of the immunobiology of ICI-treated sarcomas. The subsequent exploratory multivariate survival analyses revealed that the specific context of the immune cell composition of the TME and its interplay with neoantigens may be important for improved PFS. The insights gained from this analysis may guide the identification of prognostic biomarkers underlying sarcoma immunotherapy response and may be informative in future clinical trial designs and studies of ICI therapy in sarcomas.

## Results

### Immune cell infiltration patterns in sarcoma patients treated with ICI therapy

We first analyzed tumor expression profiles by RNA-seq of bulk tumor samples (see Methods). A principal component analysis (PCA) suggested a clustering of patients that corresponded to the sarcoma subtypes (**Supplementary Figure S1A**). Certain subtypes, such as LMS, had a notable within-subtype heterogeneity, while other subtypes such as SS, were more similar. A subsequent PCA focused exclusively on the selected collection of immune-related genes (**Supplementary Figure S1B**) suggested that a distinctive difference in immunogenomic expression profile may exist between the different sarcoma subtypes treated with ICI (**Supplementary Figure S1B**). Interestingly, two out of the three patients responding to ICI clustered together in both PCA plots.

**Figure 1:**
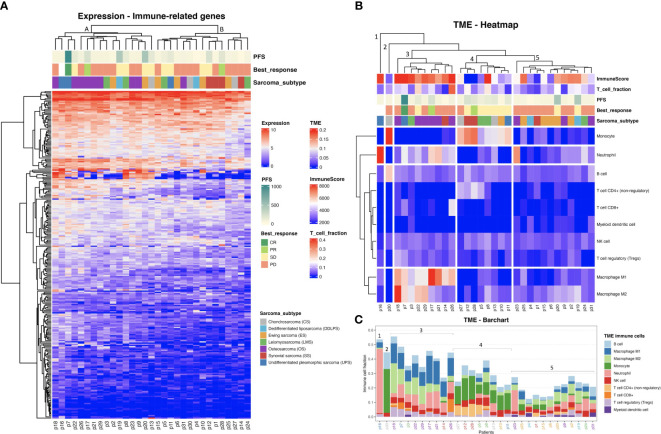
**(A)** Heat map representing the hierarchical clustering analysis of all baseline samples in two main clusters considering the expression profiles of the immune-related genes. (Progression free survival (PFS) is capped at 1000 days). **(B)** Tumor microenvironment (TME) clustering using the fraction of each immune cell type per sarcoma sample predicted by quanTIseq. ImmuneScore values are based on ESTIMATE analysis. **(C)** TME bar chart with immune cell fractions. Patient IDs are colored by sarcoma subtype as in **(A, B)**. The clusters from **(B)** are indicated.

A trend towards elevated expression of genes related to the immune response in UPS and OS relative to the other sarcoma subtypes was observed when using hierarchical clustering of immune-related genes as illustrated in **Figure 1A** (with an average transcript per million (TPM) of 94 and 84, for UPS and OS respectively, compared to the other sarcoma subtypes where the average TPM was consistently less than 60). The samples made up two main clusters: A, with almost all OS, and B, with all SS, suggesting some relation to the karyotypic subclasses. Further, a hierarchical clustering of the predicted TME composition is depicted in **Figure 1B**, and in **Figure 1C** a bar chart of the specific immune cell fractions of each cluster from **Figure 1B** is portrayed. Cluster 3 in **Figures 1B, C** consisted mostly of OS and seemed to be dominated by an elevated level of macrophages (both M1 and M2 types were detected). Interestingly, Cluster 4 was enriched in monocytes, and most of the patients in that cluster had a stable disease (SD) response to ICI therapy. Cluster 5 seemed to be the “coldest” immunological group relative to the other clusters, not surprisingly harboring most of ES samples driven by specific gene fusions. Interestingly, B cell infiltration into the TME was predicted to be a common sarcoma feature at relatively consistent levels across all sarcoma subtypes (**Figure 1C**). The limited availability of matched week-8 data from only seven patients, coupled with the absence of any observed therapeutic responses to ICI among them, hindered our capacity to infer TME composition changes following treatment (**Supplementary Figure S2**).

As observed in **Figures 1B, C**, CD8+ T cell infiltration was also predicted at varied levels among the sarcomas. The T cell infiltration predicted by quanTIseq was validated by TcellExTRECT tool. As can be observed in **Figure 1B**, TcellExTRECT results were concordant with quanTIseq, with a positive significant correlation of 0.41 and p-value = 0.022. The generic infiltration levels of non-cancer cells (*i.e.*, stroma and immune cells) across the different sarcoma samples were also evaluated using ESTIMATE toolkit. The stromal scores from ESTIMATE ranged from 1977 to 7910, while the ESTIMATE immune cell infiltration scores ranged from 574 to 9821, and finally the ESTIMATE tumor purity scores ranged from 2959 to 11086. Regarding the sarcoma subtypes, the mean and standard deviation of immune scores sorted from highest to lowest were as follows: OS, 6870 (1626); DDLPS, 6295 (2652); UPS, 60797 (2920); LMS, 5977 (2268); ES, 5832 (1884); CS, 4139 (1201); and SS, 2358 (2838). These ESTIMATE predictions corroborated the observations in **Figures 1A, B**, in that OS had a higher immune activation compared to the other sarcoma subtypes. In **Figure 1B**, the “ImmuneScore” annotation bar represents the results of ESTIMATE tool, where it can be clearly observed that Cluster 3, consisting mostly of OS, was the cluster with the highest immune scores.

Additionally, we performed a differential expression (DE) analysis comparing the patients with a clinical response to ICI (responders) to the other patients. We identified a total of 727 differentially expressed genes (DEGs), of which 209 were up- and 518 down-regulated, with an absolute fold change larger than 1 and a p-value less than 0.05 (**Supplementary Figure S3A**). Due to the small number of responders (three), multiple test correction was not applied. Enrichment analysis of the up-regulated genes revealed a significant enrichment of several immune-related Gene Ontology (GO) terms [**Supplementary Figure S3B**, corroborating the notion that an immunologically active or “hot” TME leads to improved clinical outcome to ICI therapy (17)]. A detailed table with all DEGs and the complete list of up-regulated GO terms is provided in **Supplementary Tables 1**, **2**, for DEGs and GO terms respectively. The study focuses on the immunogenomic properties of ICI-treated sarcoma patients; hence cancer hallmarks were not examined.

### Sarcoma tumors exhibit a highly heterogeneous neoantigen landscape

**Figure 2:**
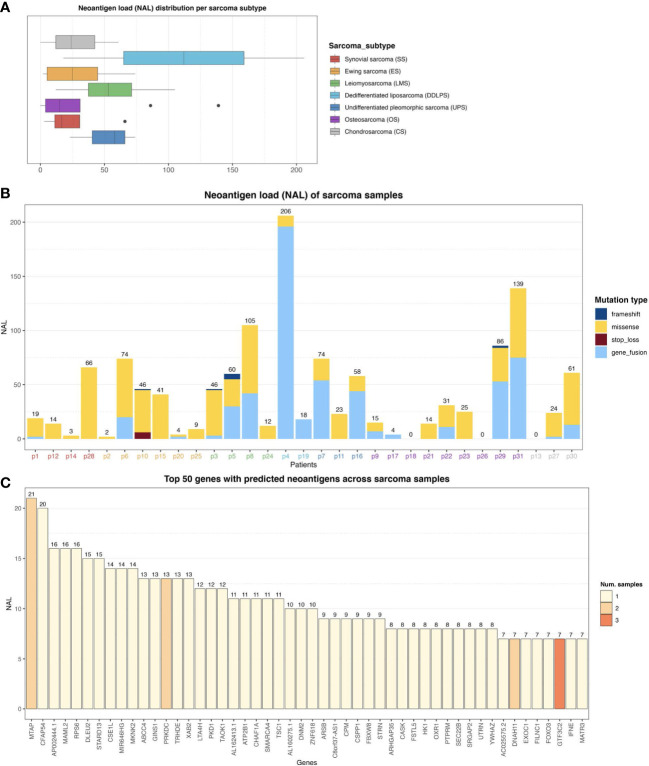
**(A)** Neoantigen load (NAL) distribution across the different sarcoma histological subtypes. The lines inside each box represents the median NAL value for each subtype while the dots are outliers. **(B)** NAL profiling of sarcoma samples and the contribution of each somatic variant type to NAL. Patient IDs are colored by sarcoma subtype as in **(A)**. **(C)** Top 50 genes with predicted neoantigens across sarcoma patients. The colors represent the number of patients with neoantigen candidates arising from the given gene.

Using a state-of-the-art somatic mutation calling framework (18), we inferred a comprehensive mutational landscape of the 31 baseline sarcoma tumor samples (see **Supplementary Figure S4** for a detailed overview). Each 9mer and 10mer peptide that had a somatic mutation was matched to the personalized HLA genotype of each individual patient to identify immunogenic neoantigens likely to be presented by the patient’s HLA alleles on their tumor cells’ surface. Such high-quality neoantigens were predicted using the NEC Immune Profiler (NIP) software (see Methods) (19), which uses an integrated artificial intelligence (AI) approach trained on proprietary data to predict antigen presentation (AP) scores, which can range from 0 to 1, for each candidate neoantigen. The distribution of neoantigen load (NAL) (see Methods) with respect to each sarcoma histological subtype was then assessed (**Figure 2A**) and revealed a highly heterogeneous NAL both between and within subtypes, ranging from 0 to 206 for intra-subtypes and a median range of 15.0 to 112.0 for inter-subtypes. DDLPS, UPS and LMS subtypes exhibited the highest NAL overall; the striking score for DDLPS is due to one case, p4, who had an extremely high number of gene fusions (**Figure 2B**). 461 (28.9%) of the mutated peptides had an AP score (see Methods) greater than 0.5 and were also predicted to bind more than one HLA allele in the same patient. **Figure 2B** shows the number of neoantigens generated in each patient, stratified by the type of somatic mutation, outlining a broad diversity of potential neoantigens detected within each subtype.

In this study, no correlation between a conventional TMB and NAL was found (Spearman Rank correlation coefficient of -0.08, and p-value of 0.66). The conventional TMB calculation typically does not take gene fusions into account, and as can be observed in **Figure 2B**, gene fusions were one of the dominant contributors to NAL for several patients. However, a clear positive correlation between the number of gene fusions and NAL was observed, with a correlation coefficient of 0.92 and p-value of 2.65e-13. We also measured the contribution of each gene to the overall NAL across all the patients. **Figure 2C** shows the top 50 most frequently mutated genes that gave rise to candidate neoantigens. For instance, MTAP gene, a methylthioadenosine phosphorylase, known to be deficient in some tumors (20), contributed with 21 neoantigens (**Figure 2C**) across two patients (**Supplementary Figure S5**). PRKDC gene, encoding for a DNA-dependent protein kinase catalytic subunit, is involved in DNA repair, the establishment of immune tolerance, and genome stability, and thus, has the potential predictive biomarker for ICI therapy (21). We detected 13 neoantigens across two patients for PRKDC gene (**Figure 2C** and **Supplementary Figure S5**). No shared neoantigens were found between the different patients, which aligns with other studies (22) and was expected here due to the highly heterogeneous mutational landscape across the sarcoma subtypes.

**Figure 3:**
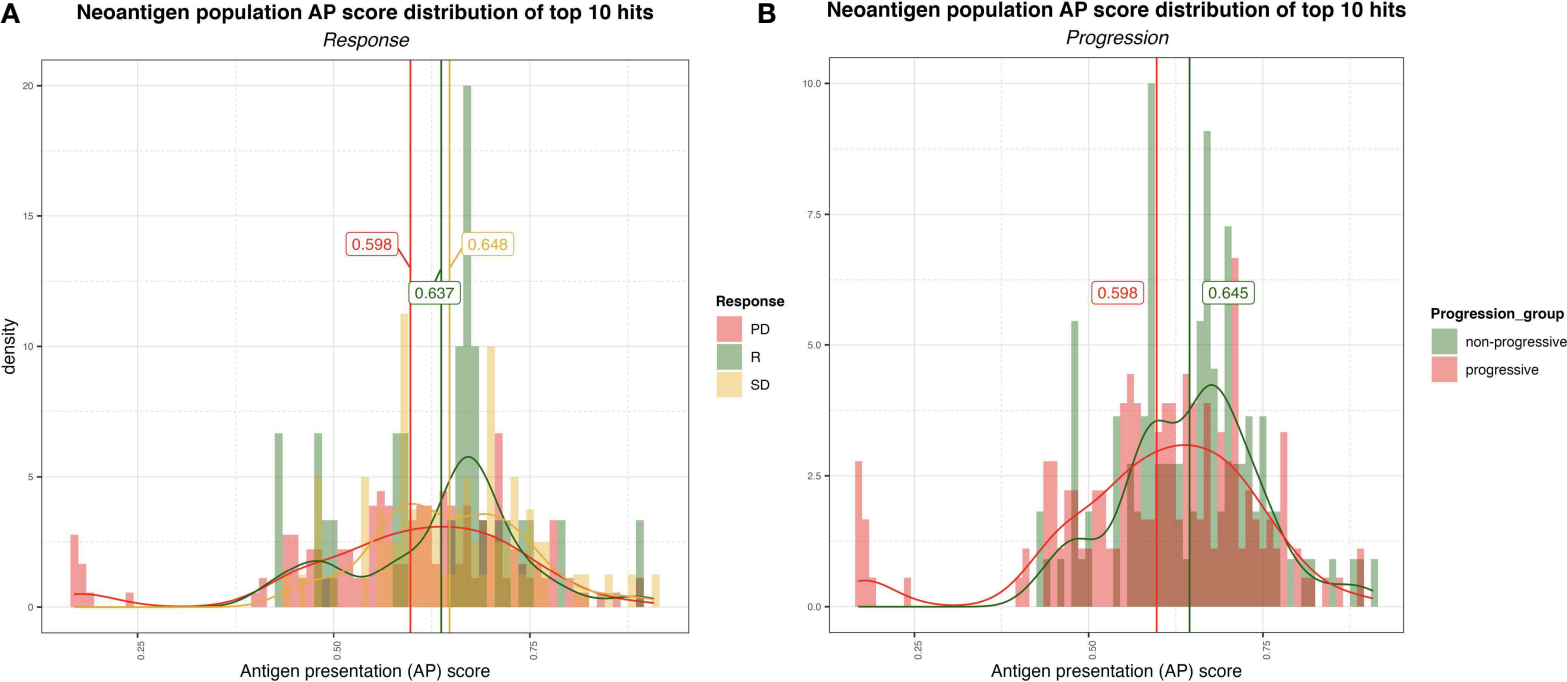
**(A)** Distribution of the AP scores for the top 10 (ranked by AP score) neoantigen candidates for progressive disease (PD), stable disease (SD) and responder (R) group. The vertical lines represent the mean AP score for each group. **(B)** Distribution of the AP scores for the top ten (ranked by AP score! neoantigen candidates for non-progressive (SD+R) and progressive (PD) groups.

We next analyzed the distribution of the ranked AP scores of the top ten neoantigen candidates for each of the baseline samples. The patients were pooled according to their clinical response, consisting of three responders, 18 patients with progressive disease (PD), and 9 patients with stable disease (SD). The distribution of AP scores, and the means of the populations of each group, were then compared using Welch’s test (**Figure 3A**). A marginally significant difference in the neoantigen scores between the responders and PD groups was observed (p-value = 0.078). There was no significant difference between the responders and SD groups (p-value = 0.63), but the comparison between the SD vs PD groups emerged with a significant difference (p-value = 0.001). Additionally, also using Welch’s test, we compared the PD group against the remaining non-PD groups (*i.e.*, SD and responders pooled together), whereby a significant difference emerged from the analysis (p-value = 0.001) (**Figure 3B**). Interestingly, this analysis revealed, on average, a higher neoantigen quality for patients without PD.

The limited number of patient samples available for analysis post ICI therapy hindered our capacity to derive significant conclusions. For the few samples that were available post therapy; none responded to ICI. In addition, removal by immunoediting of somatic mutations or immunogenic neoantigens caused by ICI therapy was not detectable among the few available ICI-treated samples (**Supplementary Figure S6**).

### Survival and clinical response of TMB, NAL and immune cell TME in ICI treated sarcomas

We next evaluated the effect of various immunogenomic features on PFS of the patients using Kaplan-Meier (KM) survival analysis. Conventional thresholds such as mean/median or Maximally Selected Rank Statistics failed to yield significant results in most cases probably due to the considerable diversity of the dataset consisting of seven sarcoma subtypes and only 31 samples. Hence, we used a supervised optimal binning approach (see Methods) to group patients into two (low and high) groups for univariate analyses (*i.e.*, each immunogenomic feature was analyzed individually); for bivariate analysis, the univariate thresholds were used to stratify patients into four groups (*i.e.*, the immunogenomic features were analyzed in pairs of low/low, low/high, high/low, high/high). We note that the small sample size available in this study may limit the robustness of the log-rank test associated with the KM analysis. We also did not account for multiple hypothesis testing in this analysis. Consequently, we do not make strong claims of statistical significance in this preliminary study. Nevertheless, this exploratory analysis does reveal features associated with differences in PFS among patient groups and which could guide further avenues for future studies.

**Figure 4:**
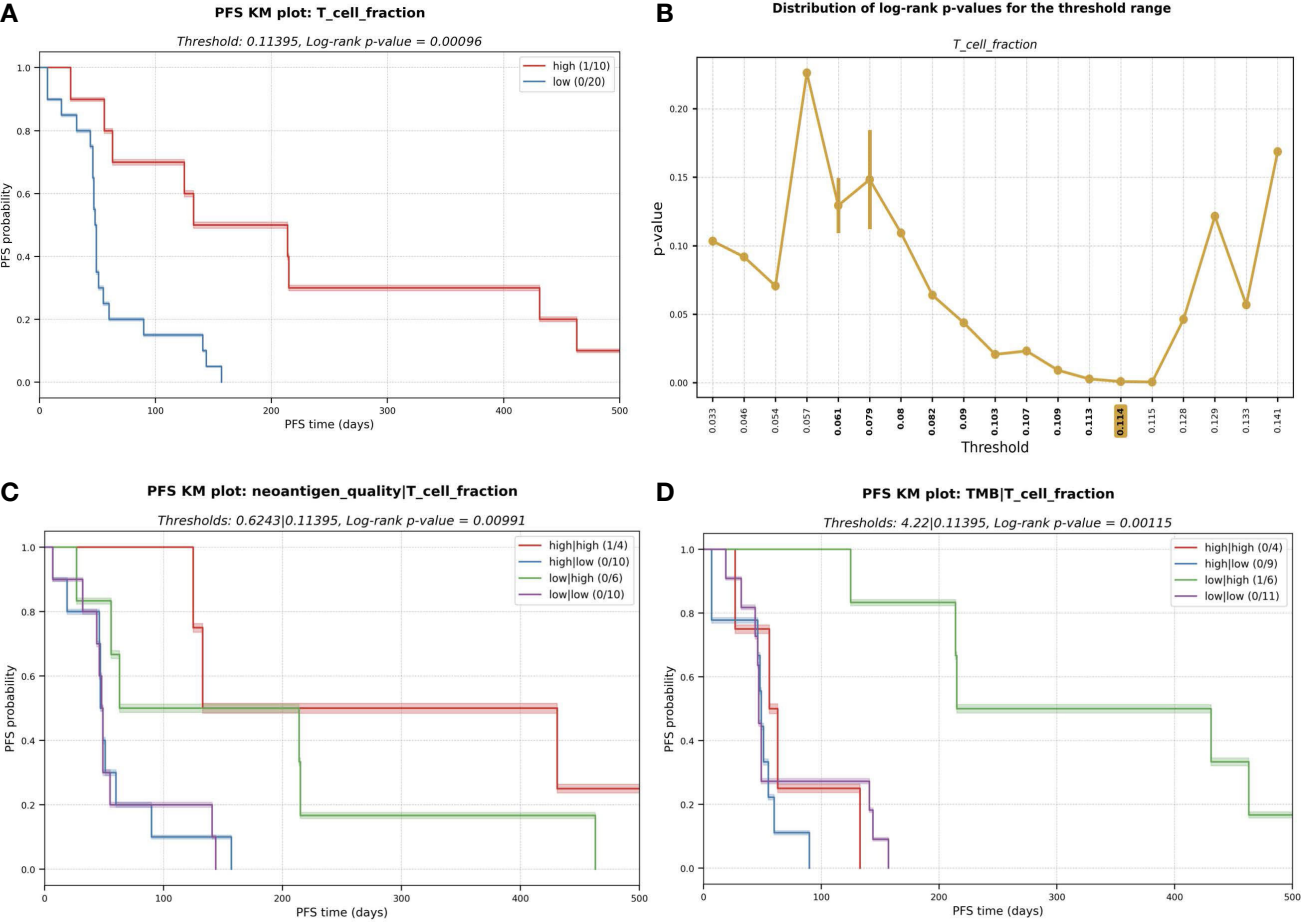
**(A)** Kaplan-Meier (KM) plot for T cell fraction univariate analysis. A higher fraction of tumor infiltrated T cell leads to better progression-free survival (PFS). PFS capped to 500 days for visualization purpose. **(B)** Distribution of log-rank p-values for the different thresholds evaluated during the exploratory supervised optimal binning approach. In bold, all the thresholds resulting in groups of at least ten patients each, among those, the one with lowest p-value (highlighted in yellow) is selected and used in **(A)**. **(C)** KM plot for the multivariate analysis with the combinatorial effect in PFS of neoantigen quality and T cell fraction; and **(D)** TMB and T cell fraction.

We first performed a statistical interrogation of the immune cell infiltration into the TME in a univariate analysis (where a supervised optimal binning approach was utilized to independently assess the effect of the considered immunogenomic features in the survival and determine the optimal threshold cutoffs, see Methods). We identified a signal that an elevated fraction of infiltrated T cells into the TME as measured by TcellExTRECT tool (see Methods), led to improved PFS with a log-rank p-value in the KM analysis of 0.00096 (**Figure 4A**). The group of patients presenting a higher proportion of infiltrated T cells had a median PFS of 173 days while the lower group had a median of 48 days. **Figure 4B** demonstrates the suitability of the supervised optimal binning approach to find the best threshold to generate the different groups evaluated in KM survival analysis. A window of low p-values across different thresholds indicates a more robust separation of low and high groups which is not sensitive to the exact chosen threshold. An improved PFS was also observed to be associated with increased levels of macrophage M1 and M2 cell infiltration (log-rank p-values 0.047 and 0.019 for M1 and M2 cells respectively, **Supplementary Figures S7A, S7B**) and associated with B cells with a log-rank p-value of 0.074 (**Supplementary Figures S7C**).

To determine the predictive potential of the effect of combining certain immunogenomic properties for the prediction of PFS, we performed multivariate analyses. We observed that a higher neoantigen quality (see Methods) combined with a high infiltrated T cell fraction was associated with an improved PFS (median of 282 days) with a log-rank p-value of 0.006 (**Figure 4C**). High T cell infiltration with low neoantigen quality was still associated with good PFS (median PFS = 139 days). However, this PFS was lower than the median PFS among all patients with a high T cell fraction (173 days); this highlights that incorporating neoantigen quality with T cell fraction improves prognostic patient groupings compared to using T cell infiltration alone. Low T cell fraction was associated with poor PFS, regardless of the neoantigen quality being high (median PFS = 49 days) or low (median PFS = 48 days; **Figure 4C**). The same association with improved PFS was not observed with TMB and T cell fraction (**Figure 4D**).

### Investigation of immune escape parameters: antigen presentation machinery, personalized HLA-typing, and the tumor-specific HLA status

**Figure 5:**
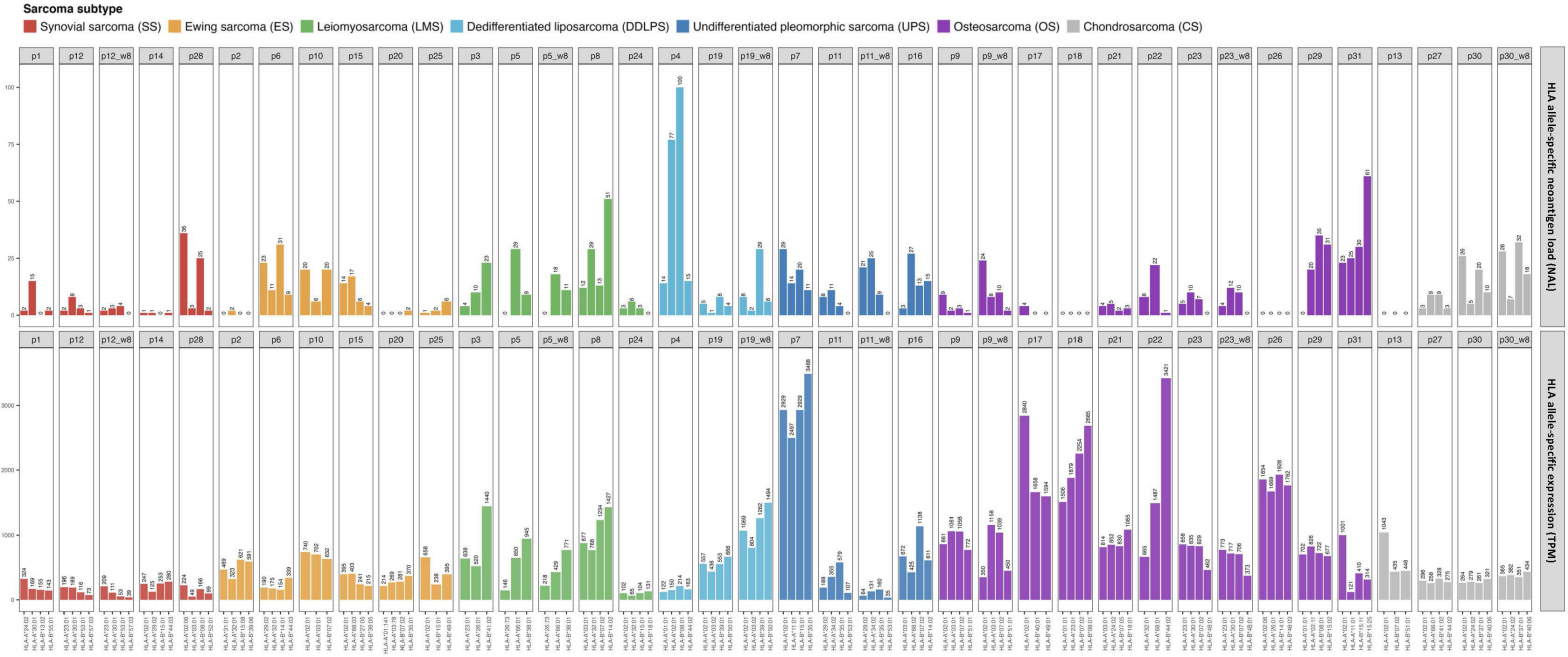
HLA-A and B allele-specific expression calculated as TPM across the different ICI-treated sarcoma samples (bottom histogram) and the correspondent NAL for each allele (top histogram).

**Figure 6:**
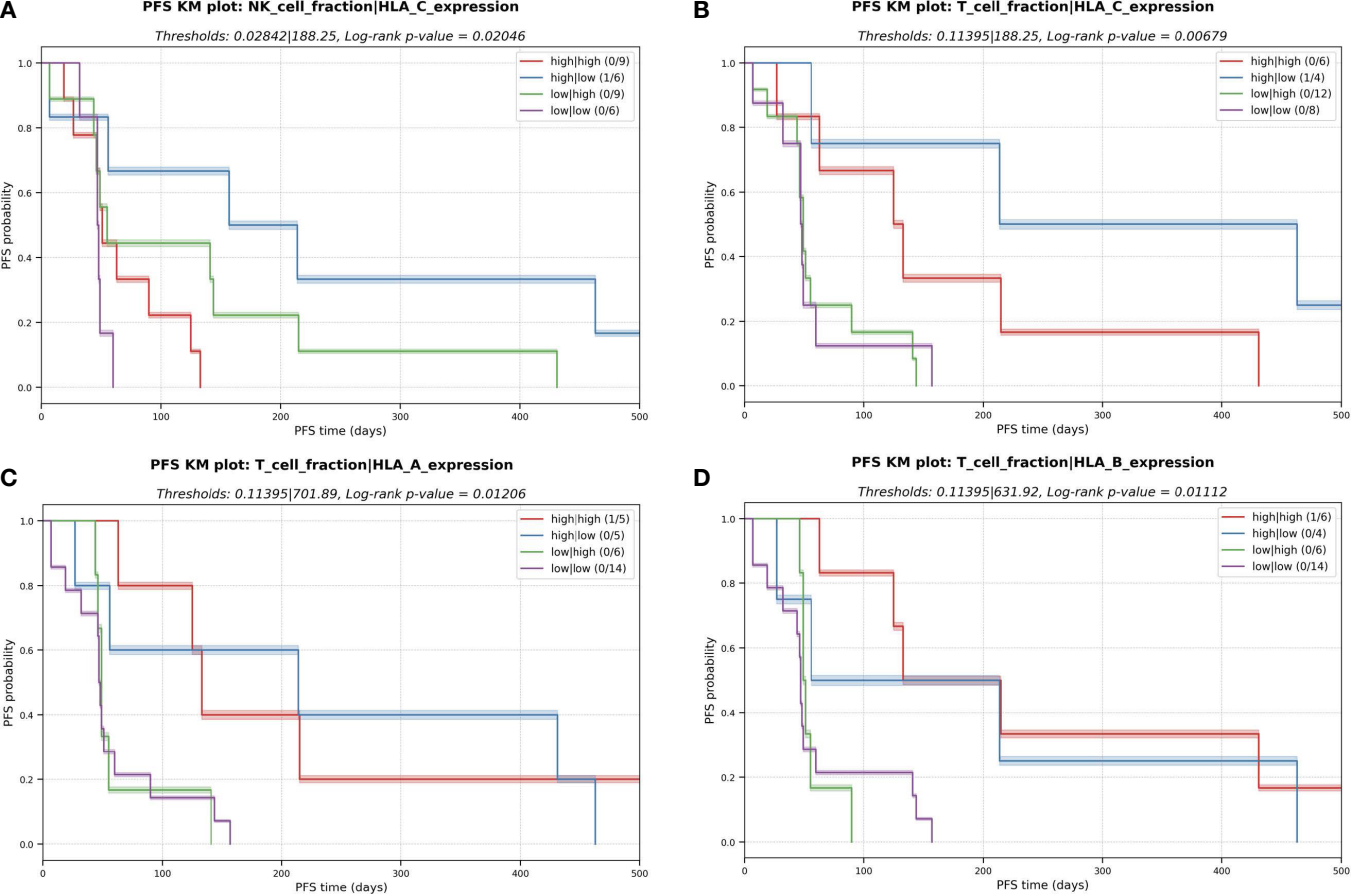
Kaplan-Meier (KM) plots for the multivariate analysis with the combinatorial effect in PFS of HLA expression and immune cell fractions in the TME. **(A)** NK cell fraction and expression of HLA-C. **(B)** T cell fraction and expression of HLA-C. **(C)** T cell fraction and expression of HLA-A **(D)** T cell fraction and expression of HLA-B.

The polymorphic nature of HLA alleles and its association to tumor-immune escape called for accurately typing and evaluating the mutation and expression status of each HLA allele in the patients (23). The status of the HLA locus in the different sarcoma subtypes was evaluated using NeoOncoHLA (24) (see Methods). Using this personalized HLA-typing approach, the somatic mutations and tumor-specific expression of each patient-specific HLA allele were described. A total of 18 somatic variants affecting HLA class I alleles were detected among 14 patients (45% of the patients presented at least one somatic mutation affecting one HLA allele). Seven of those 18 were non-synonymous variants, and six of those seven affected the peptide binding regions of the alleles. **Figure 5** depicts the expression of each HLA allele estimated as TPM and the NAL associated with each sample’s HLA-A and HLA-B alleles. The number of good neoantigen candidates for the HLA-A*02:01 allele in p9 increased from nine at baseline to 24 in week-8, while its expression (measured as TPM) decreased from 861 to 350. This could indicate a possible immune escape mechanism in this patient, through the downregulation of this HLA allele’s expression (see **Figure 5**). Although p31 showed numerous good neoantigen candidates, the expression of the HLA alleles seemed to be slightly downregulated, particularly for HLA-A*11:01. We did not consistently observe this putative immune escape pattern through HLA downregulation as a global trend across all the patients, and we had too few treated samples to draw conclusions. HLA expression did not demonstrate statistical significance in the survival study. Nonetheless, in the multivariate KM survival analysis, we found a significant separation for HLA-C (log-rank p-value = 0.001) expression in conjunction with the predicted NK cell fraction. Patients with a high NK cell infiltration combined with low HLA-C expression had the best PFS (median PFS = 157 days), while a high NK and HLA-C values resulted in short survival (median PFS = 50 days) (**Figure 6A**). This trend was not observed when combining HLA expression with T cell infiltration into the TME, where the high HLA expression and high T cell infiltration group resulted in similar PFS times as low HLA expression and high T cell infiltration group (see **Figures 6B-D**).This pattern could theoretically be owed to the combined benefit of enhanced NK cell activity due to the decreased HLA-C expression and the increased presence of T cells in the TME modulating tumor cell killing through HLA-A and/or -B antigen presentation to T cells; in the backdrop of improved PFS with high neoantigen quality with high T cell fraction reported above (see **Figure 4C**).

**Figure 7:**
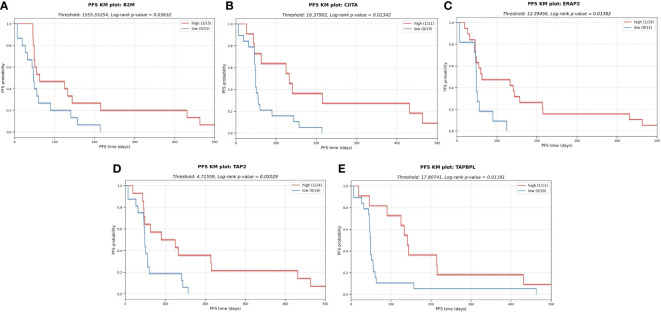
KM plots for several APM components analyzed in a univariate analysis. The downregulation of these components in the tumor cells might impair antigen presentation and subsequent escape from immune system. **(A)** Beta-2-microglobulin (B2M). **(B)** MHC class II transactivator CIITA. **(C)** Endoplasmic reticulum aminopeptidase 2 (ERAP2) **(D)** Transporter 2 (TAP2) **(E)** TAP binding protein like (TAPBPL).

Importantly, the expression profile of genes linked with the antigen presentation machinery (APM), in addition to HLA expression, revealed probable patterns of immune evasion in sarcoma. Among the APM genes we profiled (see Methods), we found that a decreased expression of beta-2-microglobulin (B2M), MHC class II transactivator CIITA, endoplasmic reticulum aminopeptidase 2 (ERAP2), transporter 2 (TAP2) and TAP binding protein like (TAPBPL) were significantly associated with a shorter PFS (**Figure 7**). Consistent with previous research, these findings underline the utility of APM profiling as a potential biomarker of tumor cell antigen presentation status and immune escape (25–27).

## Discussion

The efficacy of pembrolizumab in patients with advanced bone and soft-tissue sarcoma (STS) was assessed in the SARC028 trial (NCT02301039) and demonstrated that only some of the many different histological subclasses of sarcomas had a positive clinical response to ICI (28). In general, the results were modest, with promising results within some of the STS subtypes (6). Many studies have reported the importance of immune cell infiltration into the TME and their complex interplay with tumor cells in the clinical outcome of cancer patients (29–31). Furthermore, in recent years, neoantigens have emerged as important immunotherapy targets that play a central role in the HLA-restricted T-cell response and have been linked to the clinical efficiency of ICI therapy in several cancer indications (32, 33). Moderate responses have been found in clinical trials of ICI in sarcomas, and only a subset of patients has benefited with durable clinical outcomes (28). Due to this modest response to ICI therapy, a comprehensive immunogenomic analysis of sarcomas guided by state-of-the-art AI tools to predict neoantigens and their interplay with respect to immune cells in the TME was warranted in this study to help elucidate some of the mechanisms of possible immune escape and resistance of sarcomas to ICI therapy and to enable prior identification of patients most likely to respond.

We used a series of computational and AI methods to investigate several tumor immunogenomic properties, including the somatic mutational landscape, neoantigens, expression of key immune-related genes (e.g., APM genes), and the TME immune cell composition from the available SARC028 trial samples. The analysis was conducted on NGS data from 31 sarcoma patients, comprising WES from matched tumor and blood samples and tumor RNA-seq data. In terms of ICI response, three patients had a clinical benefit from pembrolizumab (one complete responder and two partial responders), nine had stable disease (SD), 19 had progressive disease (PD), and one did not have response information available.

Using the RNA-seq data from the tumor samples, a DE analysis revealed that several immune-related GO terms were significantly enriched in the responders, suggesting that an immunologically active TME may lead to an improved clinical response to ICI therapy. A further interrogation revealed three distinct TME immune profiles based on a hierarchical clustering of the tumor transcriptome data; (1) an immune active cluster (cluster 3), formed mostly by OS, and enriched in macrophages and CD8+ expression, (2) a cluster enriched in monocytes and with patients with SD response (cluster 4), and (3) a slightly immune desert cluster consisting of all ES patients except one (cluster 5). Importantly, two of the three ICI responders, including the patient experiencing a complete clinical response, were included in the TME immune active group (cluster 3). This was consistent with the DE analysis which showed that responders were associated with a pre-existing immune activity in the TME. The RNA-seq data was also used to deconvolute the immune cell composition in the TME, and then applied to a univariate KM survival analysis to identify immune cell infiltration patterns associated with improved PFS. We observed that higher fractions of T cells and macrophages were associated with longer PFS. These observations were consistent with previous studies that examined the importance of the immune cell composition of the TME for ICI response in sarcomas (34), although contradictory results have been reported in the literature. For example, B cell signatures correlate with longer survival times (17), whereas the converse has been reported for CD8+ T cells (35–38). Additionally, our observation of elevated macrophages in OS was consistent with previous studies (39). Similarly, using RNA-seq data, we found that a lower expression of APM-related genes, including B2M, CIITA, ERAP2, TAP2 and TAPBPL, was also associated with a shorter PFS, reflecting the increasing interest in APM biomarkers for clinical outcome and immune escape in cancer (40). Finally, RNA-seq data revealed a pattern whereby a decreased allele-specific HLA-C expression, combined with high NK cell infiltration, was linked with improved PFS. This is consistent with the well-established trend of downregulation of class I HLA allele expression correlating with NK cell activity (41).

The somatic mutation profile of the sarcomas was highly variable both within and between histological sarcoma subtypes. OS samples had the highest TMB score, followed closely by UPS, with CS having the lowest and least variable TMB. While many genes harbored mutations across the different patients, the top four most commonly mutated genes were all members of the mucin (MUC) gene family, known to play a role for epithelial tissues and reported in some neoplastic lesions (42). However, this trend may be explainable by the high degree of exon repeats in the MUC family of genes among individuals (43). In addition to small variants, copy-number variations and chromosomal rearrangements were found in many patients, including the known ES driver gene fusions of EWSR1 and SS18 genes. An interesting observation here was the high number of fusion-generated neoantigens in DDLPS compared to the other subtypes. Due to the complex karyotypes of DDLPS, it would be expected to contain many genome rearrangements, but so would OS and UPS, which did not show this pattern. This observed difference is challenging to explain but could contribute to the better response seen in patients with DDLPS compared to OS but does not explain the better response of UPS cases (30).

Increased neoantigen load (NAL) has previously been positively correlated with TMB (44, 45), and therefore described to correlate with ICI therapy response (46–48) in certain cancer indications. In this study, however, we did not find a correlation between TMB and NAL. This finding reinforces the argument that higher TMB does not necessarily always equate to higher NAL. It is important to note that the conventional TMB calculation does not consider gene fusions and therefore bypasses powerful sources of neoantigens. Additionally, the conventional TMB calculation does not consider some key determinants of antigen presentation or immunogenicity, such as antigen processing, HLA binding and the expression of the somatically mutated peptides, in addition to their distance from self (that is, the “wild type” protein).

In our univariate analysis, neither NAL nor neoantigen quality was associated with improved PFS. However, when combining neoantigen quality with the presence of T cells, we observed a striking joint behavior associated with longer PFS compared to the presence of T cells alone. When the T cell fraction was low, PFS was always low (median PFS = 48 days) regardless of neoantigen quality. This was consistent with the finding that immune T cell desert TMEs are not associated with good outcomes for ICI therapy (49). Additionally, it is reasonable that high-quality neoantigens are not effective if there are no T cells in the surrounding environment to recognize them. In the case of a high T cell count but low quality neoantigens, PFS was modestly improved (median PFS = 139 days) compared to the low T cell count patients. Remarkably, patients with both high CD8+ T cell count and high-quality neoantigens had improved PFS (median PFS=282 days). The observed improved PFS when combining the tumor infiltrated CD8+ T cell fraction and neoantigen quality was not replicated when combining T cell fraction and TMB. In this case, a good PFS was observed only for low TMB with high T cell fraction, suggesting that the quality and not quantity of neoantigens might be more relevant in certain settings for clinical benefit (50). Overall, the comparison of NAL vs TMB in the context of T cell infiltration indicated that the AI neoantigen prediction platform used to identify neoantigens is reliably predicting mutated peptides presented on the tumor cell surface that are potentially immunogenic. For B cells, a similar picture emerged, except it was required for both the neoantigen AP scores and the B cell fraction in the TME to be elevated for longer PFS. This finding is reflective of the new landscape emerging on the importance of B cell tumor infiltration, prognosis, and response to immunotherapy (17). Overall, the findings of the KM survival analysis of this study were consistent with the TME clustering which found that responders were associated with a pre-treatment, immune-inflamed TME.

The KM survival analysis based on the supervised optimal binning approach we used to arrive at the insights described in this study has several limitations and caveats. First, each set of thresholds could be considered as a hypothesis in the sense of statistical testing; appropriate multiple test corrections, such as Benjamini-Hochberg would ideally be applied if the p-value was to be interpreted for true statistical significance. In this exploratory work on a limited number of samples, though, we simply aimed to identify the best bins for the data and did not intend to make broad robust statistical claims. Thus, we believe the approach is still justified as it provides informative insights from a small patient cohort, but we acknowledge the need to validate the results in a larger patient cohort. Secondly, some choices of thresholds would have resulted in very small groups; the assumptions of many statistical tests do not hold in such cases. In this work, we limited this problem by only considering univariate thresholds which result in at least ten individuals in all groups.

In addition, it is important to note that there are several properties not considered in this study, due to the experimental data not being available for the SARC028 samples, which may also be important to help our understanding and prediction of the response to cancer immunotherapy. For example, there is increasing evidence that the gut microbiota (and related metabolites such as butyrate and cholic acid), can influence the modulation of CD8+ T cell activity and immune cell infiltration into the TME (51, 52).

In conclusion, to the best of our knowledge, this is the first study to exhaustively profile the immune cell TME of sarcomas with its interplay with immunogenic neoantigens under the context of ICI therapy, in a manner that uses advanced computational AI tools to comprehensively capture this important interplay. While the sample size for this study was small, the insights gained were suggestive that the interplay between neoantigens and immune cell infiltration patterns into the TME is a key prognostic marker of clinical response to ICI and PFS. This therefore warrants further clinical and biomarker studies with larger sarcoma cohorts.

## Materials and methods

### SARC028 trial cohort data description

The SARC028 trial (NCT02301039) recruited 86 patients. Of those, 80 patients (40 with advanced STS and 40 with bone sarcomas) were eligible for the ICI therapy (pembrolizumab, an anti-PD-1 antibody) (6). The eligibility criteria for the patients included: (a) underwent at least one previous systemic therapy; (b) metastatic STS or bone sarcoma diagnosis histologically confirmed by pathological expert in accordance with the WHO Classification of Tumors and Soft Tissue and Bone; (c) had at least one measurable lesion by RECIST 1.1 and one biopsy accessible lesion; (d) 12 years or older; (d) at least 12 weeks of life expectancy (6). STS group included the following subtypes: undifferentiated pleomorphic sarcoma (UPS), dedifferentiated liposarcoma (DDLPS), synovial sarcoma (SS) and leiomyosarcoma (LMS). The bone sarcoma group included: osteosarcoma (OS), Ewing sarcoma (ES) and dedifferentiated chondrosarcoma (CS). All the patients received intravenously a dose of 200 mg of pembrolizumab every three weeks. According to the protocol blood and tumor samples were to be collected before pembrolizumab treatment (receiving the name of “baseline” samples) and 8 weeks after the start of treatment (receiving the name of “week-8” samples). For this specific study, there were unfortunately only seven patient samples available for analysis post ICI therapy. All the research and ethical approvals and permits together with the written informed consents from all the patients were obtained prior to sample collection. The 31-sarcoma patient cohort consisted of 13 patients with STS, including four synovial sarcomas (SS), four leiomyosarcomas (LMS), two dedifferentiated liposarcomas (DDLPS), three undifferentiated pleomorphic sarcomas (UPS); and 18 patients with bone sarcomas, including six Ewing sarcomas (ES), nine osteosarcomas (OS) and three dedifferentiated chondrosarcomas.

The import and analysis of the available samples, 31 baseline and seven week-8 (**Supplementary Table 3**), to Norway was approved by the Committee for Medical Ethics in Southeastern Norway #17866. All available samples from participating centers were collected and DNA and/or RNA were purified by the trial organization and shipped to Oslo University Hospital for sequencing analysis. The data were stored and analyzed by NEC OncoImmunity in the computing infrastructure specially designed for high-level protection of sensitive personal data at the University of Oslo (53).

### Whole exome sequencing on sarcoma tumor tissue and matched PBMCs and whole transcriptome sequencing on sarcoma tumor tissue

Whole exome libraries were prepared at the Oslo University Hospital Genomics Core Facility from 100 ng of genomic DNA using the Twist Core Exome enrichment system (Twist Bioscience) following the manufacturer’s protocol. RNA sequencing libraries were constructed using the KAPA RNA Hyper kit to generate a total RNA library, which was further captured using the Twist Core Exome probe set. Variable input amounts of RNA were used depending on the availability of material (from 20-100 ng). Exomes and RNA libraries were sequenced paired-end 2x75bp using the Illumina HiSeq 4000 instrument, and FASTQ files were generated using Illumina’s bcl2fastq conversion software.

### Screening of mutational landscape of sarcoma through a comprehensive variant calling approach

The screening and characterization of single nucleotide variants (SNVs) and small insertions and deletion (indels) sculpturing the tumor genome was conducted using NeoMutate (18), a tool previously published by our group yielding very high validation rates (18). After an initial thorough inspection of the raw sequencing data, including quality control and adapter trimming, the high quality paired-end reads were mapped to the human genome (GRCh38) using BWA-MEM (54)(v0.7.17-r1188). The output BAM files were treated according to the genome analysis toolkit (GATK, v4.0.6.0) best practices (55) (including PCR duplicate marking and realignment optimization). NeoMutate incorporates an ensemble of six independent state-of-the-art somatic variant calling tools enabling the capture of the full mutational profile from tumor-normal matched WES data. Only those variant candidates detected with high confidence at least by two out of the six tools were kept avoiding false positive mutation calls. The variants were additionally filtered according to different quality thresholds (including minimum read depth (DP) of 10 reads for both tumor and normal data, more than three reads supporting the mutation in the tumor sample at the variant position). Ensembl Variant Effect Predictor (VEP) (56) toolkit was used to annotate the functional effect of the detected variants on the resulting gene product. VEP was also exploited to identify the non-synonymous variants, in other words, those mutations altering the amino acid sequence of the tumor proteome, underpinning the neoantigen landscape of tumors (see Methods section “Characterization of sarcomas immunogenicity through neoantigen prediction”). Importantly, the expression of the somatic variations was evaluated using RNA-seq data, and only the expressed somatic variants were retained for the neoantigen prediction step. This is because the altered peptides need to be expressed by the tumor to result in the production of a neoantigen.

In addition to somatic variant identification, GATK-HaplotypeCaller (v4.0.6) (57) was used for germline variant identification, and VEP (56) for the variant annotation. Importantly, the combined effect of proximal (nearby) variants (either germline or somatic) altering the same protein, and therefore, the same neoantigenic peptide, was evaluated. Haplotype phasing is the bioinformatics process of statistical estimation of haplotypes from genotype data. WhatsHap (58) (v0.17) was used giving as input both tumor WES and RNA-seq data to assess the phase relationship between proximal variants, in other words, to evaluate whether nearby variants were affecting the same allele in the same tumor subclone. After the phasing, Haplosaurus (59) (included in VEP package), was called to assess the joined functional impact of the phased variants and fully reconstruct the mutated protein sequence. Phasing step ensures that the selected neoantigen repertoire arising from the mutated proteins correctly represents the patient’s genome, increasing the chances of anti-tumor response to immunotherapy.

### Characterization of sarcoma gene fusions

It is widely recognized that large chromosomal rearrangements and fusion genes play a critical role in underpinning and driving the sarcomagenesis course in specific morphological subtypes, making them a valuable diagnostic marker (60, 61). The accurate identification of sarcoma fusion genes helps to understand its pathogenesis and the development of specific treatment strategies against the targetable fusions. In addition, gene fusions represent an incredibly valuable source of potentially immunogenic neoantigens that can mediate the anticancer immune response to ICI, even in those tumors with low TMB (62). We predicted the gene fusions from RNA-seq data using Arriba (63), the winner method for gene fusion detection in the ICGC-TCGA DREAM Somatic Mutation Calling–RNA Challenge. Arriba (63), developed for clinical research setting, is based in the ultrafast STAR aligner, and it computes a confidence score (low, medium or high) reflecting the likelihood of the fusion being generated due to an underlying genomic rearrangement specific to the tumor and not due to a sequencing artifact. Low confidence gene fusions were discarded from downstream analysis.

### Tumor mutational burden calculation

TMB was defined as the number of non-synonymous somatic SNV and indels with a VAF of at least %5 per megabase in the coding area of the cancer genome, as recommended by the guidelines of the Friends of Cancer Research TMB Harmonization Project (64). Fusions, CNVs, non-coding and synonymous mutations were discarded for TMB calculation.

### Human leukocyte antigen typing

HLA alleles of each patient were inferred in silico using OncoHLA (65) providing peripheral blood mononuclear cell (PBMC) WES data as input. OncoHLA uses an integer linear programming algorithm together with prior probabilities of the allelic ethnic frequencies to determine the closest-matched HLA allele from the IPD-IMGT/HLA Database (66) (v.3.41.2). The output includes the HLA types for both class I and class II up to four field of resolution and the associated HLA gene, transcripts, and protein sequences.

### HLA expression quantification and HLA somatic variant screening in sarcoma samples

It is well established that cancer cells can exploit several HLA-associated immune evasion mechanisms to hijack the immune system (67, 68). A comprehensive scrutiny of the HLA status in the tumor was conducted using a previously developed method by our group (24). Using the typed HLA alleles, an exhaustive profiling of the somatic mutations affecting each individual HLA allele was carried out, and their functional impact in the corresponding HLA protein sequences was annotated. In addition, the expression (abundance), reported as transcripts per million mapped reads (TPM), of each allele was quantified by mapping the RNA-seq reads to the inferred HLA sequences, to evaluate whether any allele was downregulated – a well-known immune escape mechanism in tumor development.

### Isoform and gene-level expression quantification using RNA-seq data from sarcoma samples

Gene isoform expression profiling from the RNA-seq data of both STS and bone sarcoma was carried out utilizing Kallisto (69) (v0.43.1), based on pseudoalignments of the reads and expectation-maximization (EM) algorithm to conduct isoform-level expression quantification. The reference transcriptome for GRCh38 genome, required as input, was obtained from Ensembl database version 95. Kallisto reports the abundance transcript level measured TPM.

The expression values of each transcript were used for several analyses that will be further detailed in the following sections, including: (1) Calculation of the abundance of the potential neoantigens generated from the proteins affected by non-synonymous variants in the tumor; (2) Differential expression and enrichment analysis; (3) TME profiling; (4) Manual inspection of immune-related genes expression.

### Characterization of sarcomas immunogenicity through neoantigen prediction analysis

The immunogenicity of the mutated peptides derived from tumor-specific alterations was assessed using NEC Immune Profiler (NIP) modular neoantigen pipeline developed by NEC OncoImmunity, comprising several proprietary T cell epitope machine-learning (ML) prediction algorithms. Neoantigen predictions for HLA-A and -B were conducted for each patient with peptides of lengths 9 and 10. Due to the lack of HLA-C validated data influencing the accuracy of the ML models, HLA-C was not evaluated. The pipeline considers several features determining the immunogenicity of a neoantigen, including:

1. The binding affinity of the peptide for the MHC/HLA molecule. NIP exploits three distinct binding affinity ML predictors that compute IC50 (nM) scores for each mutated peptide. The lower the IC50 score, the stronger the binding of the peptide to the specific HLA molecule.
2. The peptide’s ability to be efficiently digested by the antigen processing machinery (APM). An ensemble of 13 Support Vector Machines (SVM) included in NIP and trained on validated mass spectrometry immunopeptidome datasets determine which peptides have the optimal features to be efficiently processed by the APM, which include cleavage probability by the proteasome and antigen processing transport (TAP) efficiency.
3. The expression level of the mutated peptide. The expression of each neoantigen candidate was computed by summing the expression values (TPMs) of all the isoforms coding for the specific peptides under consideration, which is critical for the accurate prediction of immunogenic neoantigens. To determine the specific abundance of the mutated peptide, the sum of the expression levels of all the isoforms containing the peptide was adjusted according to the variant allele frequency (VAF) computed at RNA level.
4. The relative uniqueness of the candidate neoantigens compared to the normal peptides of healthy tissue was evaluated to avoid cross-reaction with self-antigen sequences.

The result is summarized in a single score ranging from 0 to 1, known as the antigen presentation score (AP), which indicates the T cell recognition probability, ranging from 0 to 1, with 1 signifying the highest likelihood that the given neoantigen is immunogenic. Neoantigen load (NAL) was computed by calculating the number of neoantigens with an AP higher than 0.5. Neoantigen quality in this study was defined as the mean AP score for the top ten neoantigen candidates ranked by AP score.

### Tumor microenvironment profiling

QuanTIseq (70) deconvolution algorithm was utilized to analyze the immune cell composition of each sarcoma sample. QuanTIseq has demonstrated a robust overall performance (71) and is one of the very few methods generating an absolute score that can be interpreted as a cell fraction, which allows both inter- and intra-sample comparisons. It takes as input RNA-seq reads and quantifies *via* deconvolution of cell fractions based on constrained least squares regression the proportion of ten different immune cell phenotypes, including B cells, M1 and M2 macrophages, CD8+, CD4+ and regulatory T cells, natural killer cells (NK), among others.

In addition to QuanTIseq, TcellExTRECT (72) R package was applied to estimate infiltrated T cell fraction. The tool employs WES data and makes use of a signal based on somatic copy number from V(D)J recombination to directly quantify the proportion of T cells.

Further, the ESTIMATE algorithm was applied to calculate the stromal and immune scores for each sarcoma sample using the normalized gene expression values as input (73).

### Selection of immune-related genes

We conducted an exhaustive literature research and compiled a list of 282 genes known to be related with immune system interaction and response (74–76). The full immune-related gene list is provided in **Supplementary Table 4**.

### Differential expression analysis and enrichment analysis

In order to characterize the differentially expressed genes (DEG) between week-8 and baseline samples, the DESeq2 (77) (v1.30.1) R package was selected. “Tximport” (78) (1.18.0) R package was utilized to import the transcript-level expression estimates generated by Kallisto and produce gene-level count matrices and normalizing offsets, as required by DeSeq2 (77). DeSeq2 (77) models the counts using normalization factors to account for differences in the library depth, estimates the gene-wise dispersions and uses shrinkage of effect size to remove the low count genes. Then, it fits a negative binomial model and performs Wald test hypothesis testing. DEGs were obtained by applying a filter of p-value<0.05.

The results of the DE analysis were expanded by conducting an enrichment analysis using an overrepresentation analysis (ORA) to associate the expression data with specific biological processes (BP). The R package called “goseq” (79) (1.42.0) conducted ORA of Gene Ontology (GO) terms, which corrects the results based on gene length bias of DEGs. DEGs were first separated into up and down-regulated genes attending to their fold change (FC) (greater than 1 or smaller than -1, for up and down-regulated genes, respectively), and then goseq (79) was used to detect overrepresented up and down-regulated BPs.

### Statistical analysis

The statistical analyses in this study were conducted using R (4.0.3) and python (3.8) programming languages. The criteria for the annotation of the different determinants of each patient to ICI therapy, such as progression-free survival (PFS) and overall survival (OS), has been previously described in (6). Survival curves were plotted using the Kaplan–Meier (KM) functionality within “lifelines” python library to compare PFS for a different set of individual covariates (univariate analysis) and in conjunction (multivariate analysis). Differences in median PFS were assessed using the log-rank test and multivariate long-rank test.

### Supervised optimal binning

Kaplan-Meier (KM) survival analysis entails splitting individuals into two or more groups and comparing survival times between individuals in the groups. For a numeric covariate, a threshold is required to perform such a split; that is, we must select a threshold to bin individuals into groups. Prior work (80) has shown that for such binning problems, the only thresholds which change group composition are the values that actually occur in the dataset. For example, when splitting individuals into groups based on TMB, the only TMB thresholds which change to which group individuals are assigned are the actual TMB values of the individuals. Thus, we determined an “optimal” threshold with respect to KM analysis by evaluating the log-rank test p-value for all possible binning of individuals in the dataset; we implemented this by simply trying each observed value for the numeric covariate of interest in the patient dataset. Only threshold cutoffs generating bins with at least 10 individuals each were evaluated.

## Data availability statement

All the datasets presented in this study are available through the SARC clinical trial data repository: https://sarctrials.org/clinical-trials/sarc028/. The data is not readily available for direct download from SARC, as it is classed as patient sensitive genetic information. However, please contact SARC at sarc028@sarctrials.org and instructions on downloading the data will be subsequently provided. The accession numbers for each of the samples used in this study can be found within the article supplementary materials (**Supplementary Table 5**).

## Ethics statement

The studies involving human participants were reviewed and approved by Regional Committees for Medical Research Ethics South East Norway. The patients/participants provided their written informed consent to participate in this study.

## Author contributions

Conceptualization, TC, OM, IA. Methodology, IA and BM. Data analysis, IA, BM, PS, BS and HF. Data investigation, LM-Z, RS, EK, MB, HT, OM, TC. Draft manuscript preparation, IA and TC. Manuscript review and editing, all authors. All authors contributed to the article and approved the submitted version.
