# Supplementary material for: The interplay between neoantigens and immune cells in sarcomas treated with checkpoint inhibition
Source: Front Immunol. 2023 Sep 20;14:1226445. doi: 10.3389/fimmu.2023.1226445 (PMC10548483; doi:10.3389/fimmu.2023.1226445)
Supplement: Supplementary file 1 [file DataSheet_1.pdf]

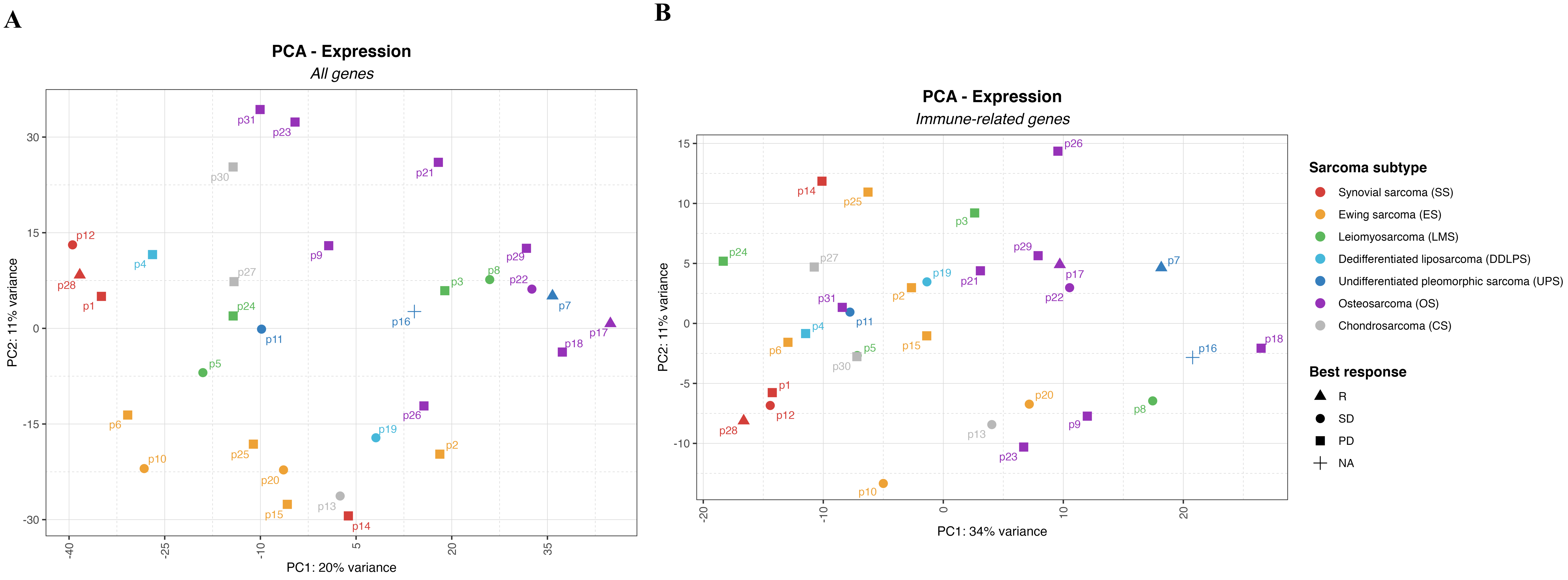

Figure S1 (A) Principal component analysis (PCA) using all the genes of the genome. **(B)** PCA using a pre-defined set of immune-related genes.
