# Supplementary material for: The interplay between neoantigens and immune cells in sarcomas treated with checkpoint inhibition
Source: Front Immunol. 2023 Sep 20;14:1226445. doi: 10.3389/fimmu.2023.1226445 (PMC10548483; doi:10.3389/fimmu.2023.1226445)
Supplement: Supplementary file 2 [file DataSheet_2.pdf]

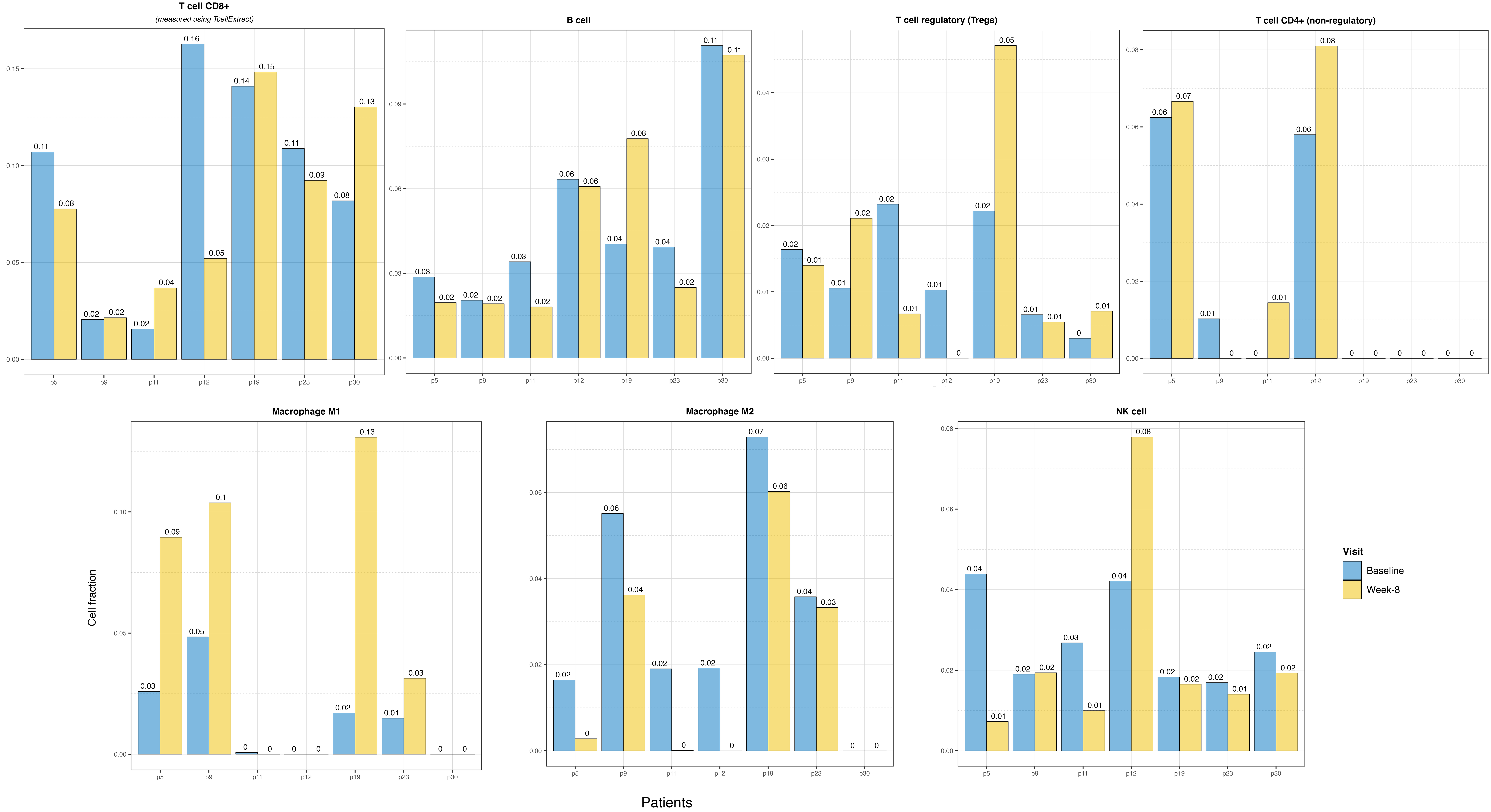

Figure S2. Change in immune cell fractions in the TME after ICI therapy. Only those seven patients with paired baseline and week-8 samples are illustrated.
