# Supplementary material for: The interplay between neoantigens and immune cells in sarcomas treated with checkpoint inhibition
Source: Front Immunol. 2023 Sep 20;14:1226445. doi: 10.3389/fimmu.2023.1226445 (PMC10548483; doi:10.3389/fimmu.2023.1226445)
Supplement: Supplementary file 3 [file DataSheet_3.pdf]

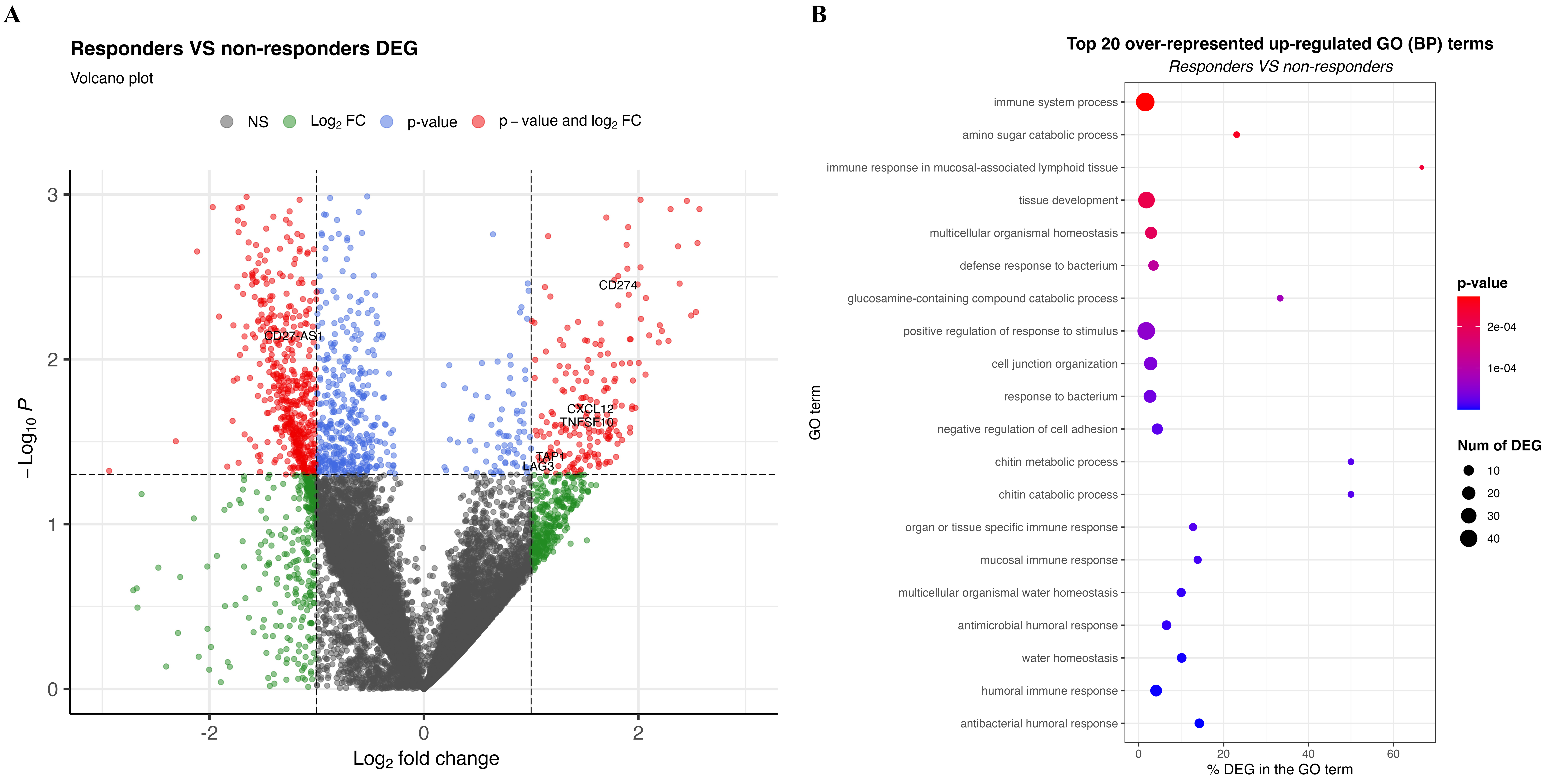

Figure S3 (A) Volcano plot summarizing differential gene expression analysis results. The plot is colored according to two variables fold-change (FC) and p-value. Genes with a p-value smaller than 0.05 and an absolute log2FC greater than 1 are consider differentially expressed genes (DEG). (grey: Not significant (NS); green: significant FC; blue: significant p-value; red: significant FC and p-value). DEGs included in the immune-related gene list are annotated. **(B)** Top 20 enriched gene ontology (GO) biological processes (BP) calculated using “goseq” and up-regulated DEGs. The x-axis represents the ratio of DEG in the term; the size of the dots represents the total number of DE genes per term; and the color indicates the p-value. Sorted by p-value.
