# Supplementary material for: The interplay between neoantigens and immune cells in sarcomas treated with checkpoint inhibition
Source: Front Immunol. 2023 Sep 20;14:1226445. doi: 10.3389/fimmu.2023.1226445 (PMC10548483; doi:10.3389/fimmu.2023.1226445)
Supplement: Supplementary file 4 [file DataSheet_4.pdf]

Heterogeneous mutational landscape among sarcoma patients

WES of both tumor and matched peripheral blood samples from the same patient was used to predict a wide catalog of genetic alterations comprising somatic point mutations (including single nucleotide variants (SNV) and small insertions and deletions (indels)) and bigger structural rearrangements (such as copy number (CN) alterations and gene fusions)). The number of variants was quite diverse both within and between subtypes, suggesting the presence of the two molecularly distinct sarcoma categories, those driven by simple and complex karyotypic defects. TMB metric, calculated based on the detected set of non-synonymous small somatic point mutations (see Methods), confirmed the observed mutational heterogeneity, resulting in a TMB ranging from 0.95 to 13.59 and with an inter-subtype median range of 2.29-6.38 (Figure S4A). Notably, in OS and SS subtypes, the TMB distribution showed outliers with substantially higher TMB values (Figure S4A), reflecting the intra-subtype variability underlying tumor biology for these subtypes. This genetic variability was also observed with respect to gene fusion incidence, with an inter-subtype median range of 8.0-78.0 (Figure S4B). Patient p4 presented 151 gene fusions, however, due to the low number of patients within DDLPS (only two), it is difficult to assess if this is an outlier. Moreover, the variants were classified with respect to their functional effect in the resultant gene product (including frameshift, inframe, missense, variants affecting the splice sites, variants affecting the start and stop codons, fusion events, and copy number alterations leading to gain or loss of copies) and their specific incidence on each patient was described (Figure S4C). We also investigated the mutational rate of each gene across the different sarcoma samples and the landscape of mutations affecting them (Figure S4D). Interestingly, we observed a high mutation rate across several members belonging to the mucin (MUC) gene family, known to play a role function for epithelial tissues and reported in several neoplastic lesions[1,2], including sarcoma[3,4]. Furthermore, we explored the somatic mutational profile of those genes known to be related to cancer reported in OncoKB database[5]. Multiple cancer genes were found recurrently mutated (Figure S3e), including ARID1A tumor suppressor, mutated in 64.52% of the patients and MBD6 gene, and 45.16% of the patients with gene MBD6 mutated, mostly by frameshift variants, which are associated with cancer progression[6]. Several patients also presented severe aberrations (frameshift, gene fusions, and copy number alterations) in STAT5B, TGFBR2 and DAZAP1 (Figure S4D). In addition, we studied the recurrence of non-synonymous somatic small variants between the different patients, resulting in 81.88% unique variants present in a single patient and 18.12% recurrent variants shared by at least two patients. Interestingly, MUC3A, was not only the gene mutated in the highest frequency among the patients (Figure S4D), but also the exact mutations altering it are recurrent among several patients. EWSR1/FLI1 gene fusion was reported in COSMIC[7] database and a known driver in ES sarcomagenesis[8,9] was detected in 50% of ES patients (see the frequency of gene fusions events affecting EWSR1 gene in Figure S4E). Moreover, four recurrent fusions shared between two patients, HEY1/NCOA2, SS18/SSX1, AC131392.1/AC146944.2 and CFAP44/AC112128.1 were reported, the first two also known in COSMIC[7] and related with sarcomagenesis, while the last two were formed mostly by non-coding RNAs. The genes most commonly affected by a fusion event, regardless of the specific fusion pair, were SS18 and EWSR1 (n=4), KANSL1 (n=3) and TPH2, THSD4 and MTAP (n=2).

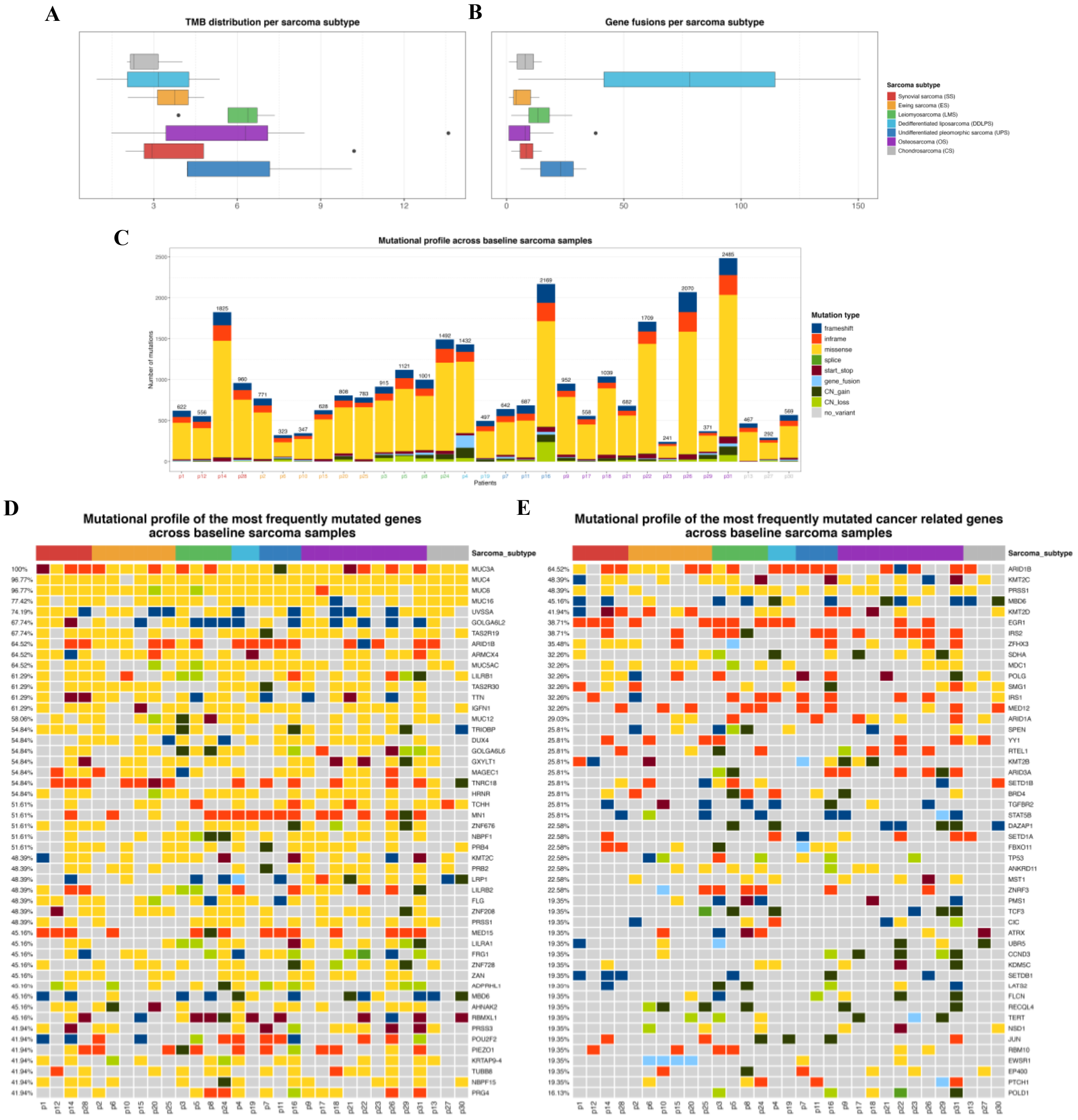

Figure S4. Mutational profile of baseline (pre-treatment) sarcoma samples. **(A)** Mutational profile of baseline sarcoma samples with the total amount of mutations categorized in different variant types according to their functional annotation, including frameshift and inframe indels,, missense SNVs, variants affecting the splice sites, variants affecting the start and stop codons, fusion events and copy number (CN) alterations leading to gain or loss of copies. **(B)** TMB distribution across the different sarcoma histological subtypes. The lines inside each box represents the median TMB value for each subtype while the dots are outliers. **(C)** Gene fusion distribution across the different sarcoma histological subtypes. **(D)** Mutational profile of the most frequently mutated genes across the sarcoma samples (capped to top 50). Each row represents a gene and each column a patient. On the left the mutation frequency for each gene is showed. Columns are ordered according to sarcoma subtype and rows according to mutation frequency. **(E)** Mutational profile of the most frequently mutated cancer related genes compiled from the OncoKB database (capped to top 50).

1. Nakamori, S., Ota, D.M., Cleary, K.R., Shirotani, K. & Irimura, T. MUC1 mucin expression as a marker of progression and metastasis of human colorectal carcinoma. *Gastroenterology* **106**, 353-361 (1994).
2. Deng, J., *et al.* The role of tumour-associated MUC1 in epithelial ovarian cancer metastasis and progression. *Cancer Metastasis Rev* **32**, 535-551 (2013).
3. Jiang, L., *et al.* Differential gene expression identifies KRT7 and MUC1 as potential metastasis-specific targets in sarcoma. *Cancer Manag Res* **11**, 8209-8218 (2019).
4. Liu, W., *et al.* Integrated genomic and transcriptomic analysis revealed mutation patterns of de-differentiated liposarcoma and leiomyosarcoma. *BMC Cancer* **20**, 1035 (2020).
5. Chakravarty, D., *et al.* OncoKB: A Precision Oncology Knowledge Base. *JCO Precis Oncol* **2017**(2017).
6. Choi, Y.J., Yoo, N.J. & Lee, S.H. Mutation and expression of a methyl-binding protein 6 (MBD6) in gastric and colorectal cancers. *Pathol Oncol Res* **21**, 857-858 (2015).
7. Tate, J.G., *et al.* COSMIC: the Catalogue Of Somatic Mutations In Cancer. *Nucleic Acids Res* **47**, D941-D947 (2019).
8. Kallen, M.E. & Hornick, J.L. From the ashes of "Ewing-like" sarcoma: A contemporary update of the classification, immunohistochemistry, and molecular genetics of round cell sarcomas. *Semin Diagn Pathol* **39**, 29-37 (2022).
9. Grunewald, T.G., *et al.* Chimeric EWSR1-FLI1 regulates the Ewing sarcoma susceptibility gene EGR2 via a GGAA microsatellite. *Nat Genet* **47**, 1073-1078 (2015).
