# Supplementary material for: The interplay between neoantigens and immune cells in sarcomas treated with checkpoint inhibition
Source: Front Immunol. 2023 Sep 20;14:1226445. doi: 10.3389/fimmu.2023.1226445 (PMC10548483; doi:10.3389/fimmu.2023.1226445)
Supplement: Supplementary file 5 [file DataSheet_5.pdf]

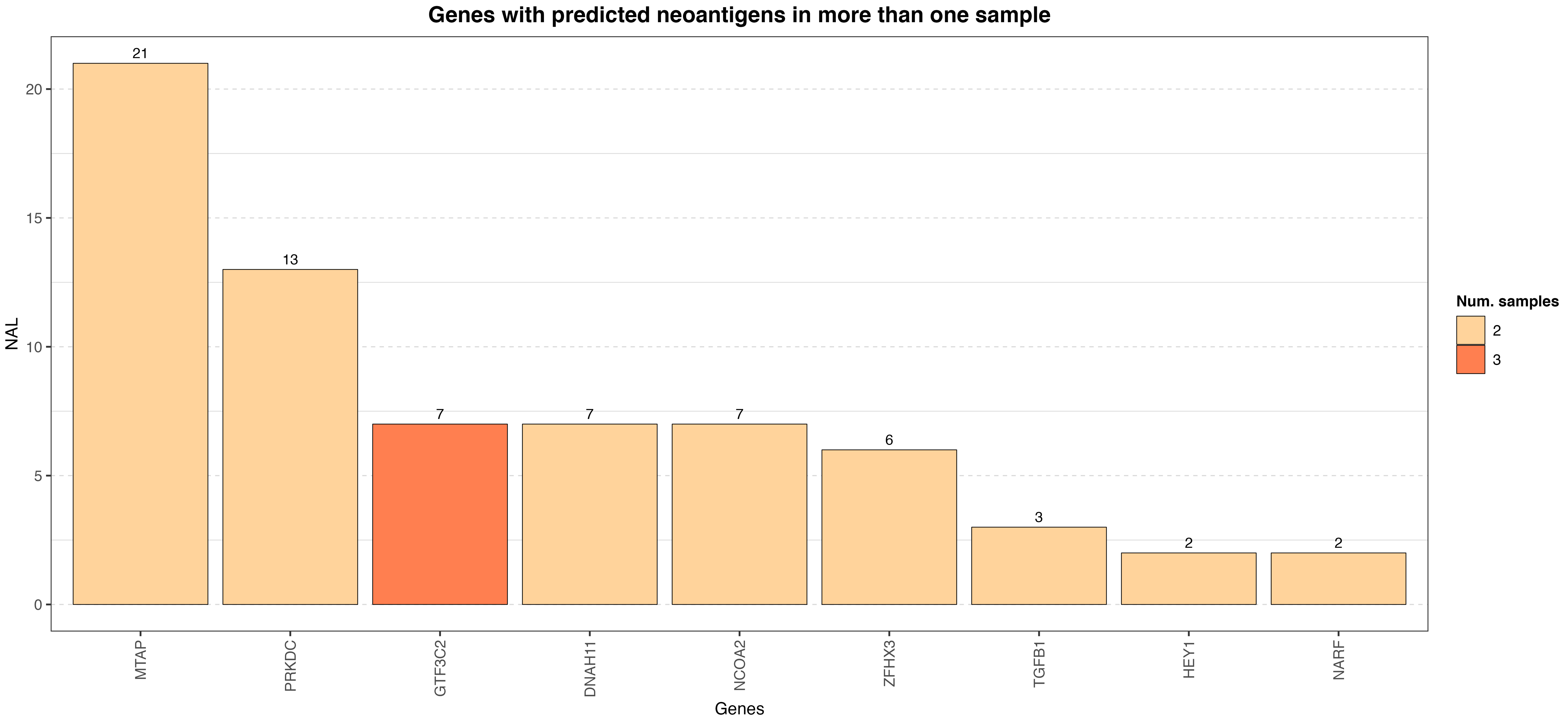

Figure S5. Mutated genes responsible of the generation of neoantigen candidates in at least two patients.
