# Supplementary material for: The interplay between neoantigens and immune cells in sarcomas treated with checkpoint inhibition
Source: Front Immunol. 2023 Sep 20;14:1226445. doi: 10.3389/fimmu.2023.1226445 (PMC10548483; doi:10.3389/fimmu.2023.1226445)
Supplement: Supplementary file 6 [file DataSheet_6.pdf]

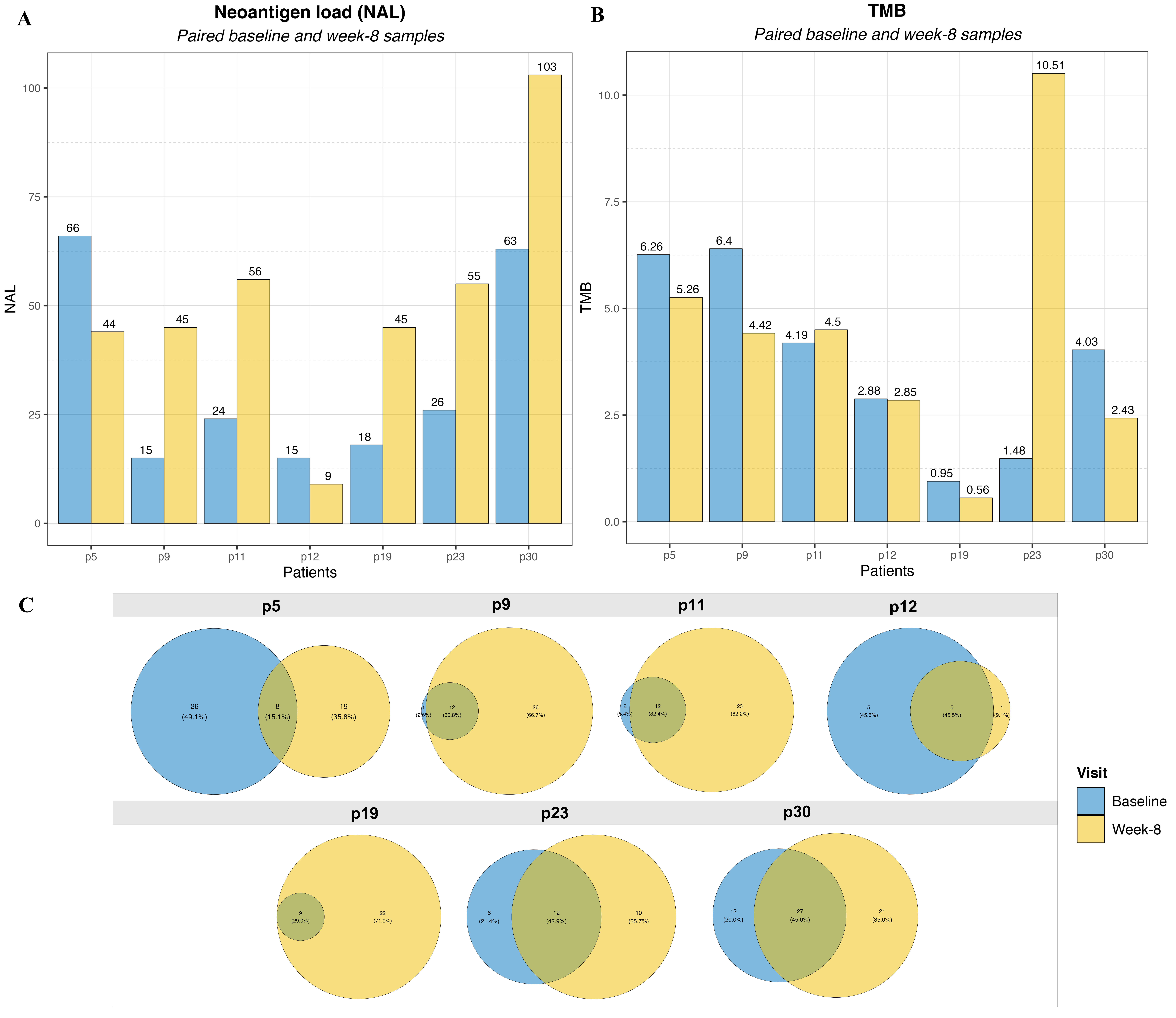

Figure S6. General overview of the mutational and neoantigen profiles for the paired baseline and week-8 after ICI treatment samples. **(A)** Neoantigen load (NAL). **(B)** Tumor mutational burden (TMB). **(C)** Venn diagrams illustrating the neoantigen overlap between baseline and week-8 samples.
