# Supplementary material for: The interplay between neoantigens and immune cells in sarcomas treated with checkpoint inhibition
Source: Front Immunol. 2023 Sep 20;14:1226445. doi: 10.3389/fimmu.2023.1226445 (PMC10548483; doi:10.3389/fimmu.2023.1226445)
Supplement: Supplementary file 7 [file DataSheet_7.pdf]

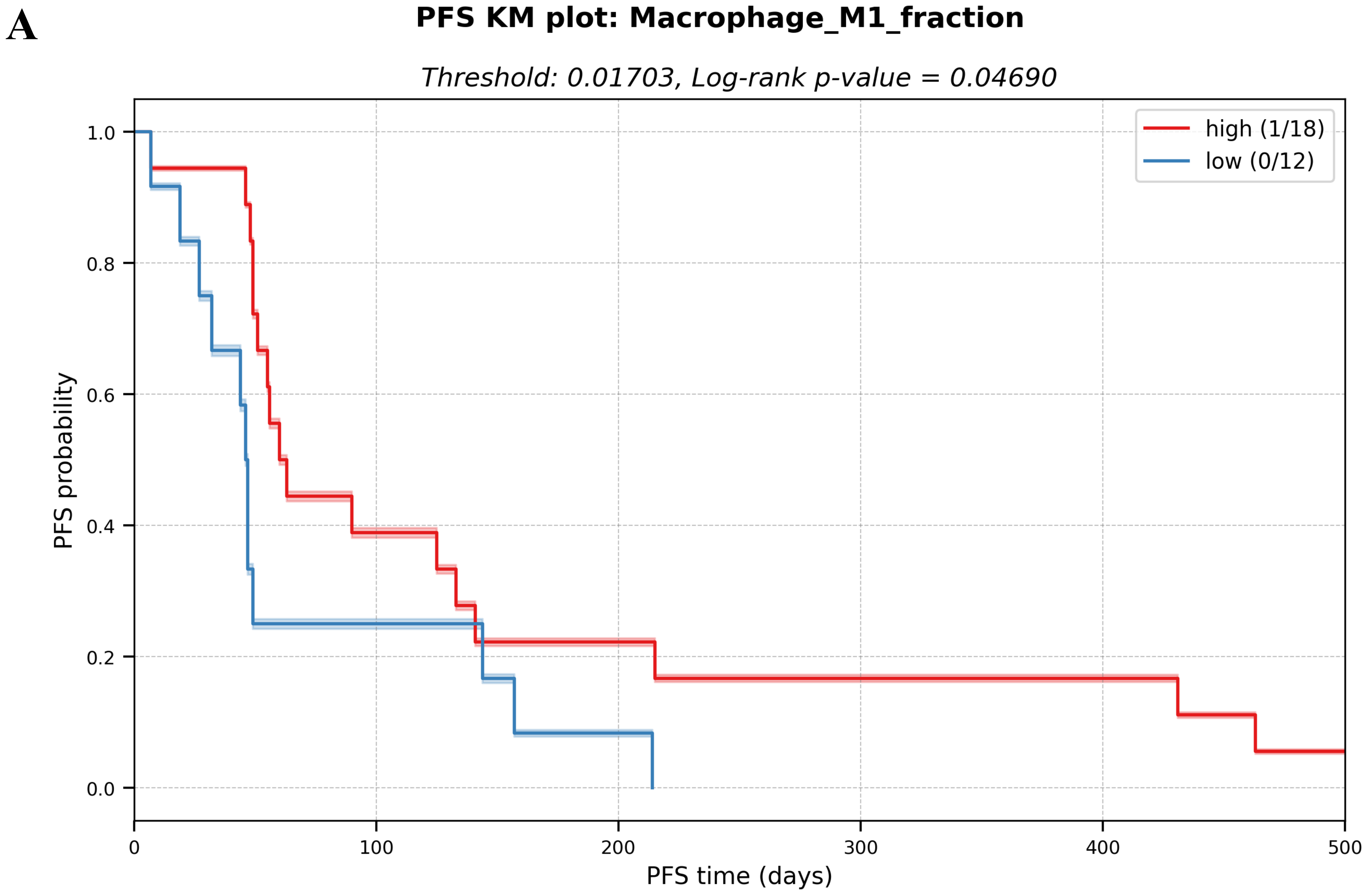

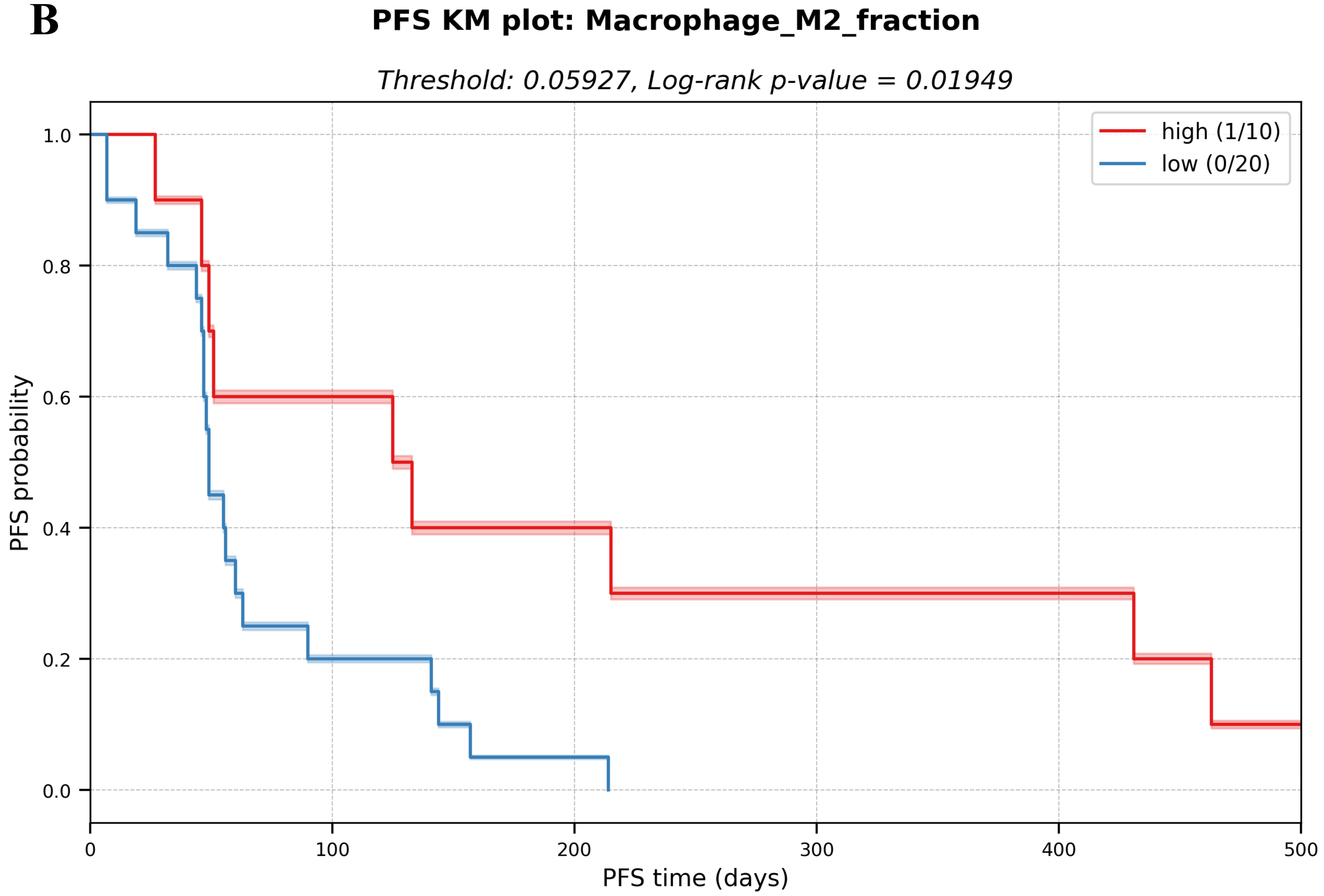

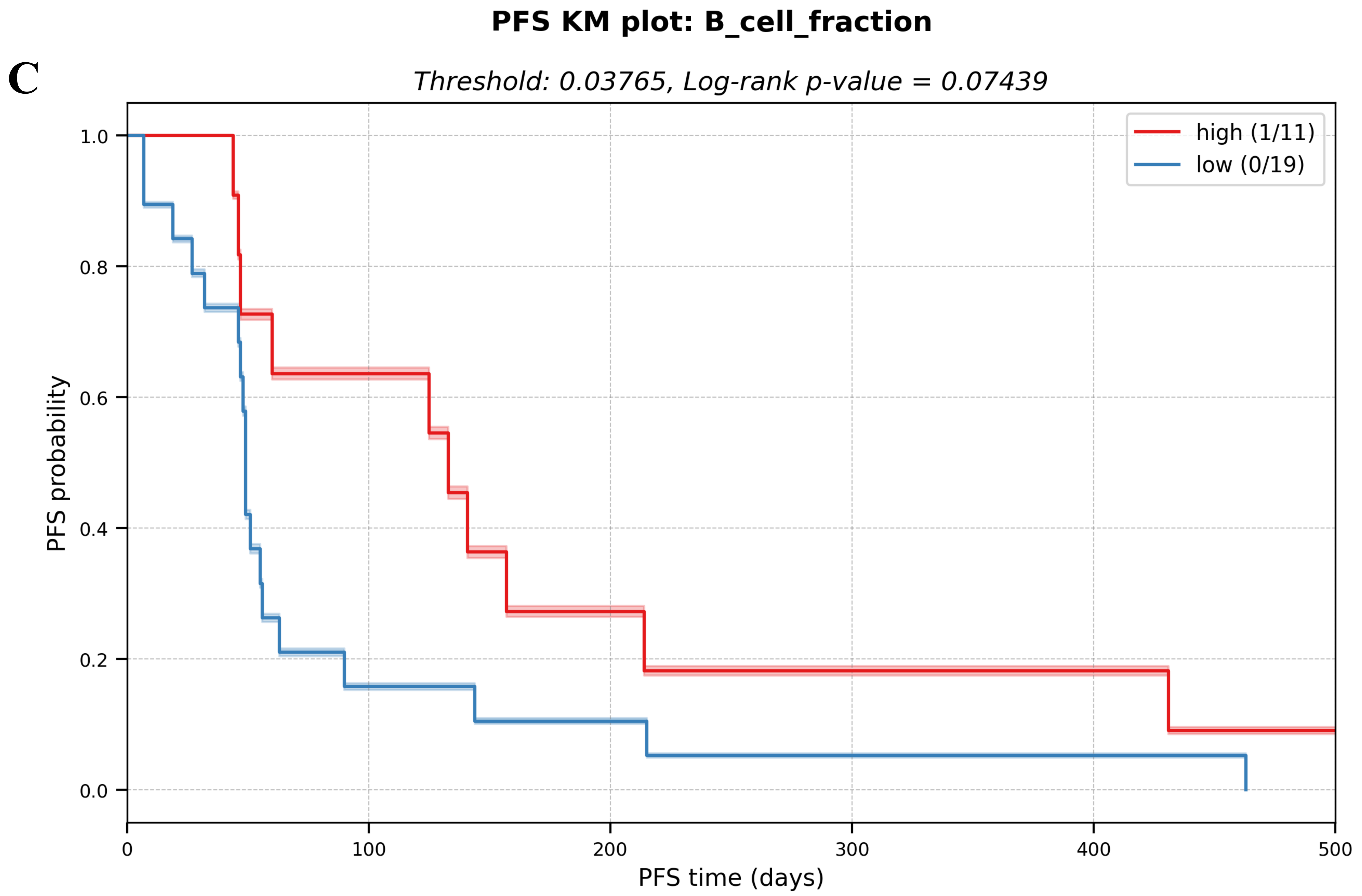

Figure S7. Kaplan-Meier (KM) plots for the univariate analysis of immune cell fractions in the TME **(A)** Macrophage M1 cell fraction. **(B)** Macrophage M2 fraction. **(C)** B cell fraction
